# Direct Observation of Protein Microcrystals in Crystallization Buffer by Atmospheric Scanning Electron Microscopy

**DOI:** 10.3390/ijms130810553

**Published:** 2012-08-22

**Authors:** Yuusuke Maruyama, Tatsuhiko Ebihara, Hidetoshi Nishiyama, Yuji Konyuba, Miki Senda, Takuro Numaga-Tomita, Toshiya Senda, Mitsuo Suga, Chikara Sato

**Affiliations:** 1 Biomedical Research Institute, National Institute of Advanced Industrial Science and Technology (AIST), Higashi 1-1-1, Tsukuba, Ibaraki 305-8566, Japan; E-Mails: yuusuke-maruyama@aist.go.jp (Y.M.); t.ebihara@aist.go.jp (T.E.); 2 Advanced Technology Division, JEOL Ltd., Akishima, Tokyo 196-8558, Japan; E-Mails: hinishiy@jeol.co.jp (H.N.); ykonyuub@jeol.co.jp (Y.K.); msuga@jeol.co.jp (M.S.); 3 Structure Guided Drug Development Project, JBIC Research Institute, Japan Biological Informatics Consortium (JBIC), Aomi 2-4-7, Koto-ku, Tokyo 135-0064, Japan; E-Mail: miki-senda@aist.go.jp; 4 National Institute of Environmental Health Sciences–NIH, Department of Health and Human Services, 111 TW Alexander Dr, Research Triangle Park, NC 27709, USA; E-Mail: tomitat2@niehs.nih.gov; 5 Biomedicinal Information Research Center, National Institute of Advanced Industrial Science and Technology (AIST), Aomi 2-4-7, Koto-ku, Tokyo 135-0064, Japan; E-Mail: toshiya-senda@aist.go.jp

**Keywords:** X-ray crystallography, protein crystal, nano-crystal, crystallization screening, environmental cell, TAF-Iβ, micro-focus X-ray beams, X-ray free-electron laser, ASEM, ClairScope

## Abstract

X-ray crystallography requires high quality crystals above a given size. This requirement not only limits the proteins to be analyzed, but also reduces the speed of the structure determination. Indeed, the tertiary structures of many physiologically important proteins remain elusive because of the so-called “crystallization bottleneck”. Once microcrystals have been obtained, crystallization conditions can be optimized to produce bigger and better crystals. However, the identification of microcrystals can be difficult due to the resolution limit of optical microscopy. Electron microscopy has sometimes been utilized instead, with the disadvantage that the microcrystals usually must be observed in vacuum, which precludes the usage for crystal screening. The atmospheric scanning electron microscope (ASEM) allows samples to be observed in solution. Here, we report the use of this instrument in combination with a special thin-membrane dish with a crystallization well. It was possible to observe protein crystals of lysozyme, lipase B and a histone chaperone TAF-Iβ in crystallization buffers, without the use of staining procedures. The smallest crystals observed with ASEM were a few μm in width, and ASEM can be used with non-transparent solutions. Furthermore, the growth of salt crystals could be monitored in the ASEM, and the difference in contrast between salt and protein crystals made it easy to distinguish between these two types of microcrystals. These results indicate that the ASEM could be an important new tool for the screening of protein microcrystals.

## 1. Introduction

The determination of three-dimensional structures is necessary for understanding protein functional mechanisms and for strategic drug design. Methods such as X-ray crystallography [1–3], nuclear magnetic resonance [4], and electron microscope single particle reconstruction [5–7] have been used for this purpose. In X-ray crystallography, once large (typically more than 50–100 μm in length) and well-ordered crystals are obtained, crystal structure determination is relatively easy with synchrotron radiation. However, determining appropriate crystallization conditions remains a bottleneck due to multiple parameters, such as concentrations of proteins, salts and precipitants, pH, and temperature.

Optical microscopy (OM) has been widely used for screening of crystallization conditions, however light wavelength restricts the resolution of diffraction-limited OM. Furthermore, for precise observation using optical microscopes, crystallization solution in a plastic screening plate should be transferred to a glass support with more optical-uniformity to observe birefringence. In addition, it is usually difficult to distinguish protein and salt crystals by OM. If micro-crystals of a target protein can be detected in solution, crystallization conditions can be optimized from a very early stage of screening. A high-throughput screening method with micro-crystal observations could therefore reduce the time necessary for obtaining crystals suitable for the crystallographic analysis. Moreover, micro-focus X-ray beams [8] has opened a new possibility to determine the protein structure using small crystals around 10 μm in size [9], and X-ray free-electron laser develops a novel method to determine the crystal structure using nano-crystals [10]; the need for a high-resolution visualization method for protein micro-crystals in solution is increasing.

Traditional electron microscopy (EM), requiring the sample to be in vacuum, can observe protein crystals using pretreatments such as freeze-etching or replica formation [11,12]. However, because handling delicate crystals is difficult and time consuming, EM has rarely been used to screen protein crystallization conditions.

**Figure 1:**
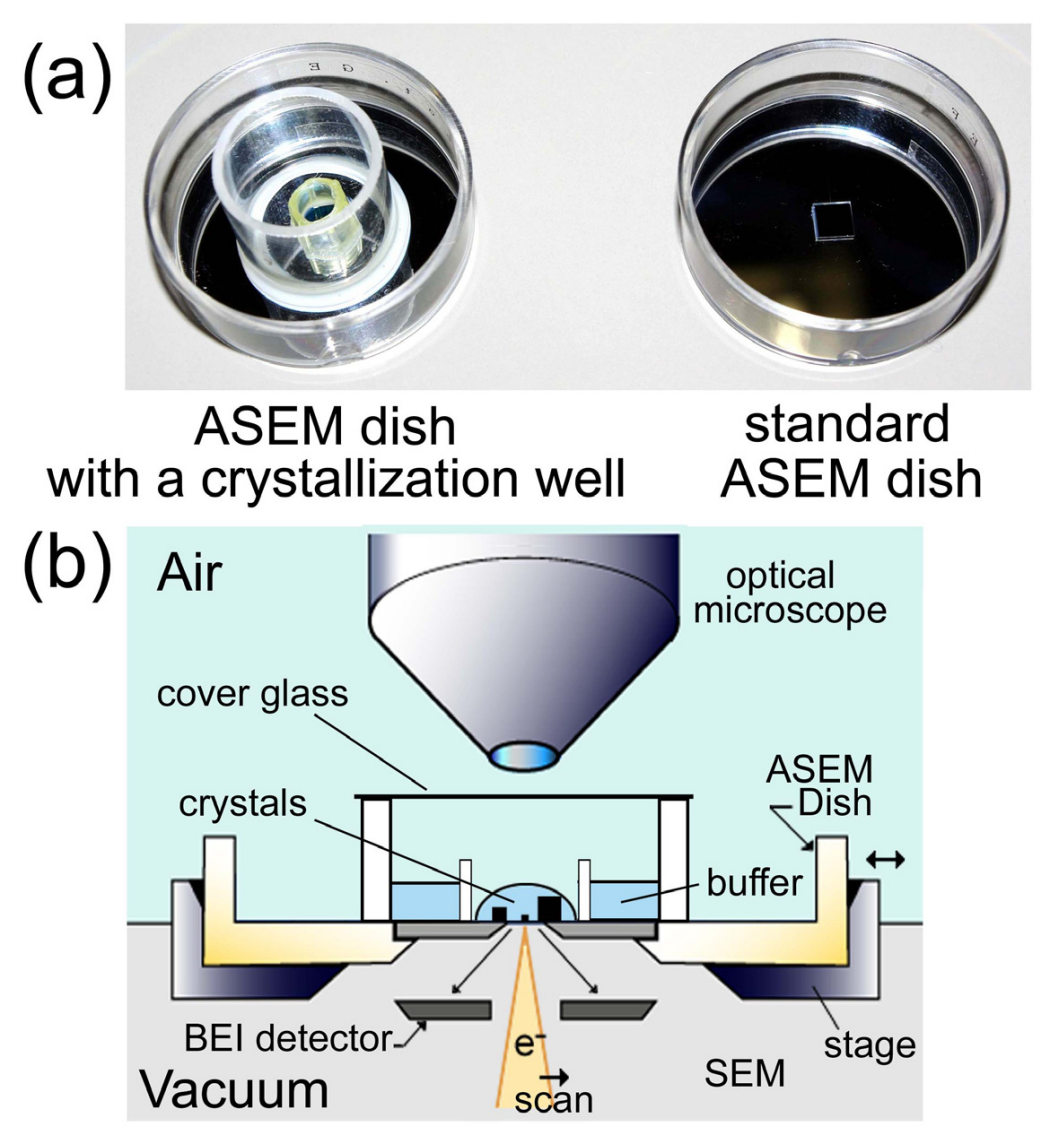
Schematic diagram of the atmospheric scanning electron microscope (ASEM). (**a**) A standard ASEM dish and an ASEM dish with a well for the protein crystallization. A removable 35-mm ASEM dish has a 100 nm silicon nitride (SiN) film window separating vacuum and sample (right). For protein crystallization, an ASEM dish with a crystallization well was prepared (left). The capacity of the well is the same as that in the standard crystallization plate for the protein crystallography; (**b**) Schematic diagram of ASEM using the ASEM dish with the crystallization well. The electron beam is projected from underneath onto crystals in buffer, through the electron-transparent film. Backscattered electrons are captured by the backscattered electron imaging (BEI) detector. An optical microscope (OM) is arranged above/opposite the inverted SEM. The optical axes of both microscopes are aligned to observe the same area, with a sample stage that can shift two-dimensionally.

EM observation of samples in solution has been accomplished using environmental cells with electron-transparent thin-film windows [13,14], which are constantly improving. Building on thin-film technology, the new Atmospheric Scanning Electron Microscope (ASEM) can visualize samples in solution at 8 nm resolution [15]. A detachable open dish sits atop an inverted SEM; the electron beam scans samples in solution through its pressure-resistant thin-film window (Figure 1). The Backscattered Electron Imaging (BEI) detector is located beneath the membrane, while an OM observes the sample from above. Since ASEM achieves fast, high resolution, and in-solution observation, ASEM seems to be well suited to protein crystal observation.

Here, we video-recorded copper sulfate crystallization in solution, utilizing the open sample holder of ASEM. Since the amount of backscattered electrons are related to atomic numbers and densities of atoms in the sample, we used heavy-metal staining to visualize lysozyme micro-crystals (2 × 2.5 μm), which are difficult to identify using OM. Finally, we directly observed non-stained crystals of three different proteins in crystallization buffers.

## 2. Results and Discussion

### 2.1. Observation of Salt Crystals and Their Growth

**Figure 2:**
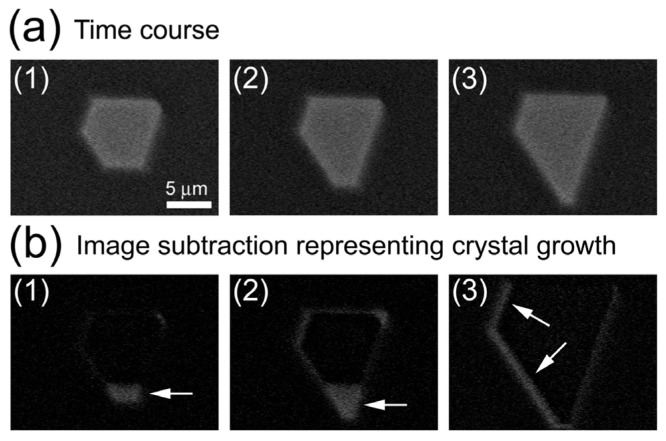
Crystal growth of copper sulfate observed with ASEM. (**a**) Images captured from ASEM video recording of crystallization in a saturated copper sulfate solution (1)–(3). A bright area, interpreted to be a copper sulfate pentahydrate crystal, gradually grows due to evaporation and temperature decrease. Time interval is 60 s. (**b**) Growth of copper sulfate crystal. Growth is demonstrated by subtracted images of **b**(1) and **b**(2), which correspond to **a**(2) minus **a**(1) and **a**(3) minus **a**(1), respectively. Bright areas indicated by arrows in **b**(1) and **b**(2) show anisotropic growth. A subtracted image **b**(3), 224 s after **a**(1) minus 216 s after **a**(1), shows rapid growth over only 8 s. The original video images can be seen in Figures S1 and S2.

To confirm that the ASEM can visualize crystals in liquid, we observed the formation of copper sulfate crystals. Since copper sulfate crystals contain enough amounts of heavy metals, they would give a significant contrast, suggesting that these are suitable samples for the first crystal observation by ASEM. The ASEM dynamically visualized salt formation due to evaporation of phosphate buffered saline [16]. Taking advantage of the open ASEM dish configuration, warm copper sulfate solution was partially evaporated and cooled on the dish, under video observation at ×5000 magnification (Figure S1). Images captured at every 60 s are shown in Figure 2a. A bright hexagonal object with parallel opposite sides grew during observation; it was later determined by OM to be a blue copper sulfate pentahydrate crystal. Crystal growth was highlighted by subtracting images in Figure 2b. Figure 2b(1) is a subtracted image of Figure 2a(2) minus Figure 2a(1), and Figure 2b(2) is that of Figure 2a(3) minus Figure 2a(1). Arrows indicate the main areas of growth: maximum outward growth was about 2.0 μm in Figure 2b(1), and 3.9 μm in Figure 2b(2). Other areas grew less than 0.5 μm during this 120-s observation, exhibiting anisotropic growth. Figure 2b(3) shows growth of crystal at later time course from 216 to 224 s after Figure 2a(1). In the 8 s, the crystal grew rapidly by about 0.84 μm as indicated by arrows. This implies that later growth could be faster than early growth. The full video between 210 and 230 s after Figure 2a(1) is shown (Figure S2). Image comparison between OM and ASEM and crystal growth observation by ASEM clearly showed that ASEM could be utilized for crystal observation in solution.

**Figure 3:**
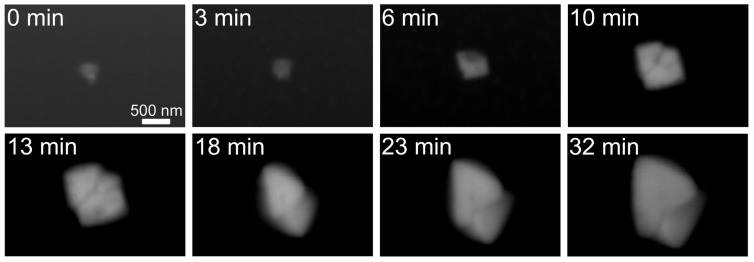
Growth of a copper sulfate crystal smaller than the wavelength of light. A 300 nm copper sulfate crystal was monitored using time-lapse imaging. Values on the top left indicate elapsed time.

We next tried to observe a smaller copper sulfate crystal which was difficult to detect using OM (Figure 3, top left). A 300 nm crystal grew in an anisotropic manner about five times larger over 32 min (Figure 3, bottom right). The observation of the crystal growth of copper sulfate revealed that each crystal face has its specific growth rate and the growth rate of each crystal face was not constant. These characteristics of crystal growth may be attributable to dislocation, random surface adsorption, lattice strain, deformation and/or physical incorporation of impurities [17].

### 2.2. Metal Staining of Protein Crystals

**Figure 4:**
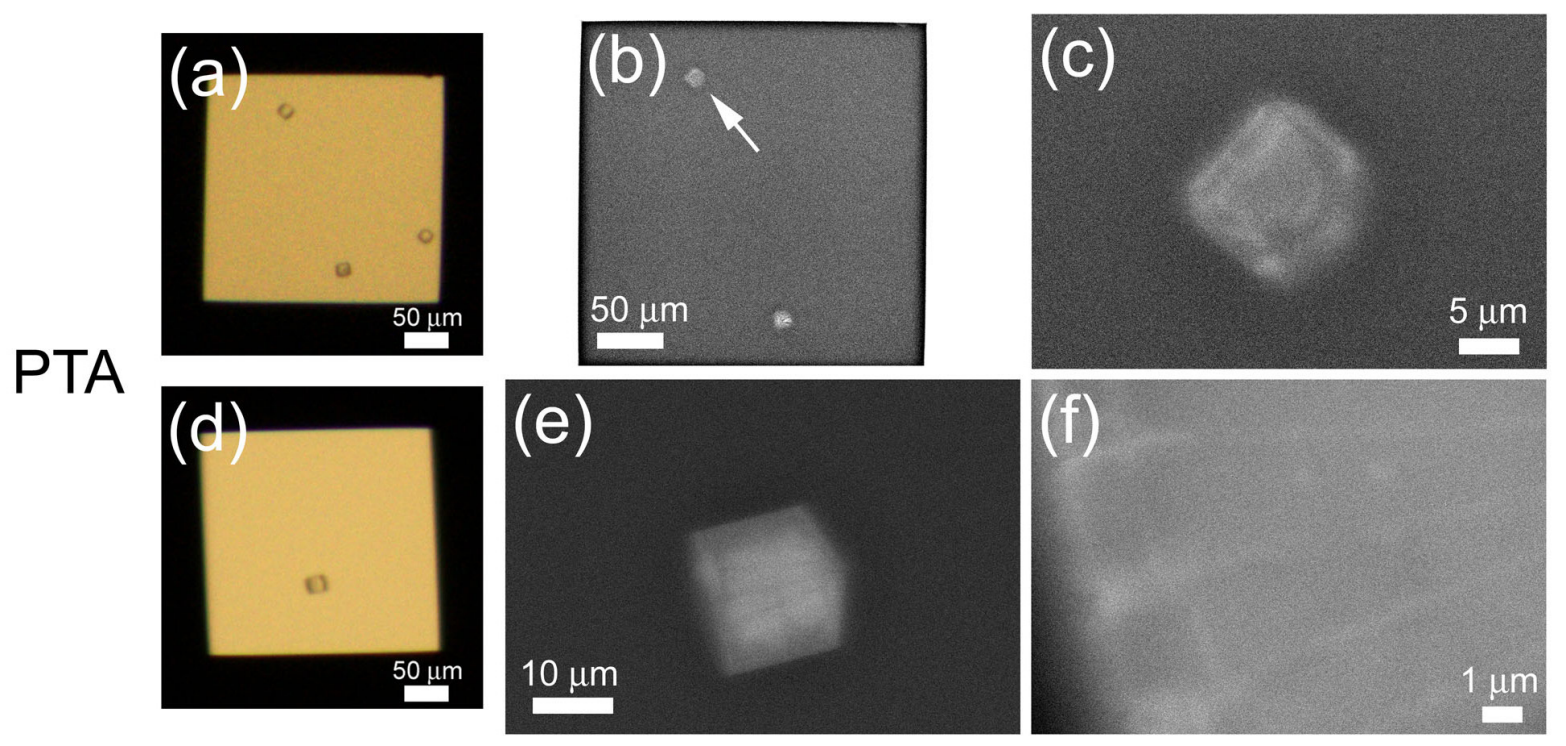
In-solution electron microscopy (EM) of lysozyme crystals stained with phosphotungstic acid solution. A lysozyme crystal on the ASEM dish was directly monitored using an optical microscopy (OM) (**a**,**d**). Crystals were stained with 1% phosphotungstic acid (PTA). Low-magnification ASEM images (**b**) correspond well to OM images (**a**), except a missing crystal on the right of (**b**), due to washing. A crystal indicated by an arrow (**b**) is further magnified (**c**). At higher magnification, the internal structure can be observed (**f**).

Next, we tried to observe protein crystals in solution. Tetragonal lysozyme crystals were formed on the ASEM dish and observed using OM (Figure 4a,d). Generally, in SEM observation, the amount of backscattered electrons is related to the sample’s atomic number and density; target with large atomic number or high density is observed in white. Considering average atomic number and density of proteins, protein crystals are likely to be observed slightly white above the darker background of water solution. To increase contrast, phosphotungstic acid (PTA) staining is adapted to lysozyme crystals. After washing, crystals in the washing buffer were visualized by ASEM as white polygons (Figure 4b,c); outlines correspond well to the OM image (Figure 4a). The tetragonal lysozyme crystal has four hexagonal faces {110} and eight quadrilateral faces {101}. Four faces {101} are adjacent to each other and form a pyramidal structure in the crystal; sides of the pyramid are four hexagonal faces {110} [18]. Therefore, the view in Figure 4c appears to be a perspective from the direction of {101}. Another view has a pentagonal outline (Figure 4e), while OM showed an ambiguous tetragonal outline due to resolution limits (Figure 4d). The thick center of the crystal appears white, the thin periphery dark. The blurred edges of the crystal could reflect their greater distance from the silicon nitride (SiN) membrane [15]. Higher magnification (×10,000) of the left edge of the crystal revealed a regular partition-like structure (Figure 4f). During PTA staining, a lysozyme crystal was rinsed away from the membrane (Figure 4b compared with Figure 4a).

### 2.3. Staining of Lysozyme Micro-Crystals

**Figure 5:**
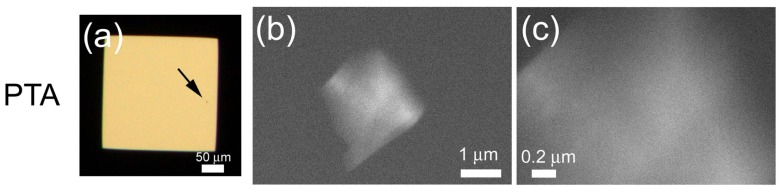
Lysozyme micro-crystals stained with PTA. After OM (**a**), lysozyme crystals were stained with 1% PTA. At high magnification, a micro-crystal (approximately 2 × 2.5 μm) (a; arrow) was observed clearly (**b**,**c**).

To examine the capability of ASEM for protein micro-crystal observation, we next tried to observe micro-crystals of lysozyme around 1 μm. Immediately after the detection of micro-crystals using OM (Figure 5a), crystal growth was stopped with the washing buffer. After staining, the ASEM visualized a tetragonal 2 × 2.5 μm crystal, at ×20,000 (Figure 5b) and ×60,000 (Figure 5c) magnification. The crystal has a dense center, stained white.

**Figure 6:**
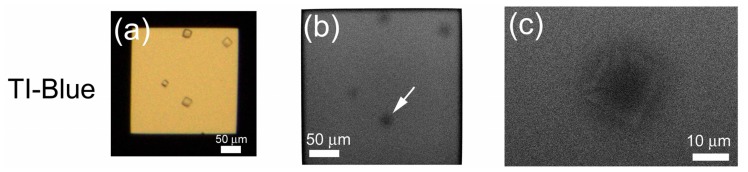
Lysozyme crystals stained with TI-Blue. Lysozyme crystals observed with OM (**a**) were stained with 0.3% TI-Blue, and observed using the ASEM (**b**,**c**). A crystal indicated by an arrow (**b**) is further magnified (**c**). TI-Blue scarcely stains protein (compare to the non-stain in Figure 7).

In contrast to the PTA staining, crystals stained with TI-Blue appear black at low magnification (×330) (Figure 6b). At higher magnification (×2000), the crystal periphery appears white (Figure 6c), but the image is much fainter than those stained with PTA, reflecting the weak staining preference of TI-Blue for proteins. Accordingly, PTA is more favorable as the standard stain for protein.

### 2.4. Non-Stained Lysozyme Protein Crystal in Crystallization Buffer

**Figure 7:**
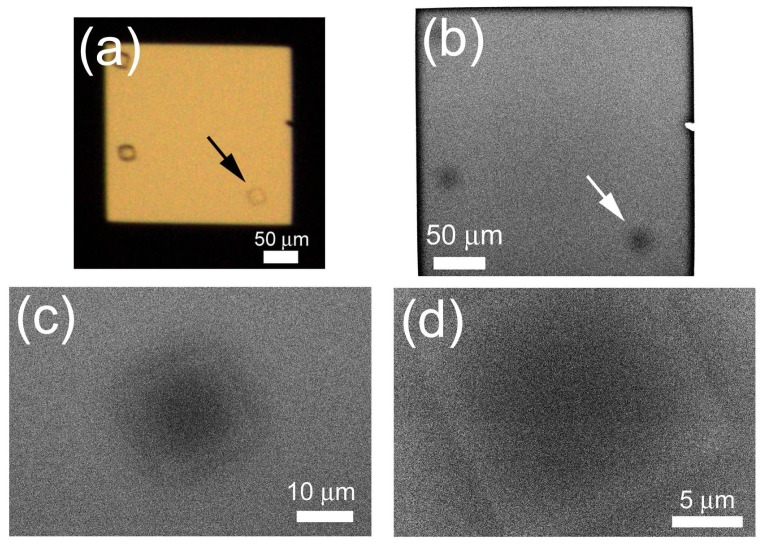
Non-stained lysozyme crystals in crystallization solution. Lysozyme crystals were observed using OM (**a**) and inverted SEM (**b**–**d**). An arrow indicates a magnified crystal. The shape of the crystal is clear, although the images are noisy and of low contrast.

Although PTA staining increases contrast, staining reduces experimental throughput and may dissolve protein crystals. For quick observation in a natural state, we tried *in-situ* observation of non-stained protein crystals. After a lysozyme crystal was confirmed using OM (Figure 7a), the crystal was observed using the ASEM at different magnifications (Figure 7b–d). The crystal was readily visible, with a white periphery and dark center (Figure 7d). Although the ASEM image is noisy and of low contrast, it is clear that the crystal is an oblique square in shape, agreeing with the OM image.

### 2.5. Observation of Non-Stained TAF-Iβ Crystal

**Figure 8:**
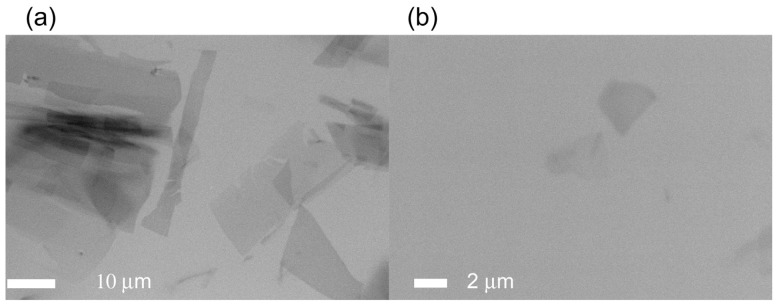
Non-stained TAF-Iβ crystals from droplet solution. A few μm-crystals of TAF-Iβ were observed using inverted SEM (**a**,**b**). The images feature multi-layer of thin crystals with trapezoid or triangle outlines (**a**); while some of the crystals appeared in the monolayer state (**b**). The images are noisy and of low contrast. The smallest observable dimension is a few μm in ASEM (compare these with OM image in Figure 9).

**Figure 9:**
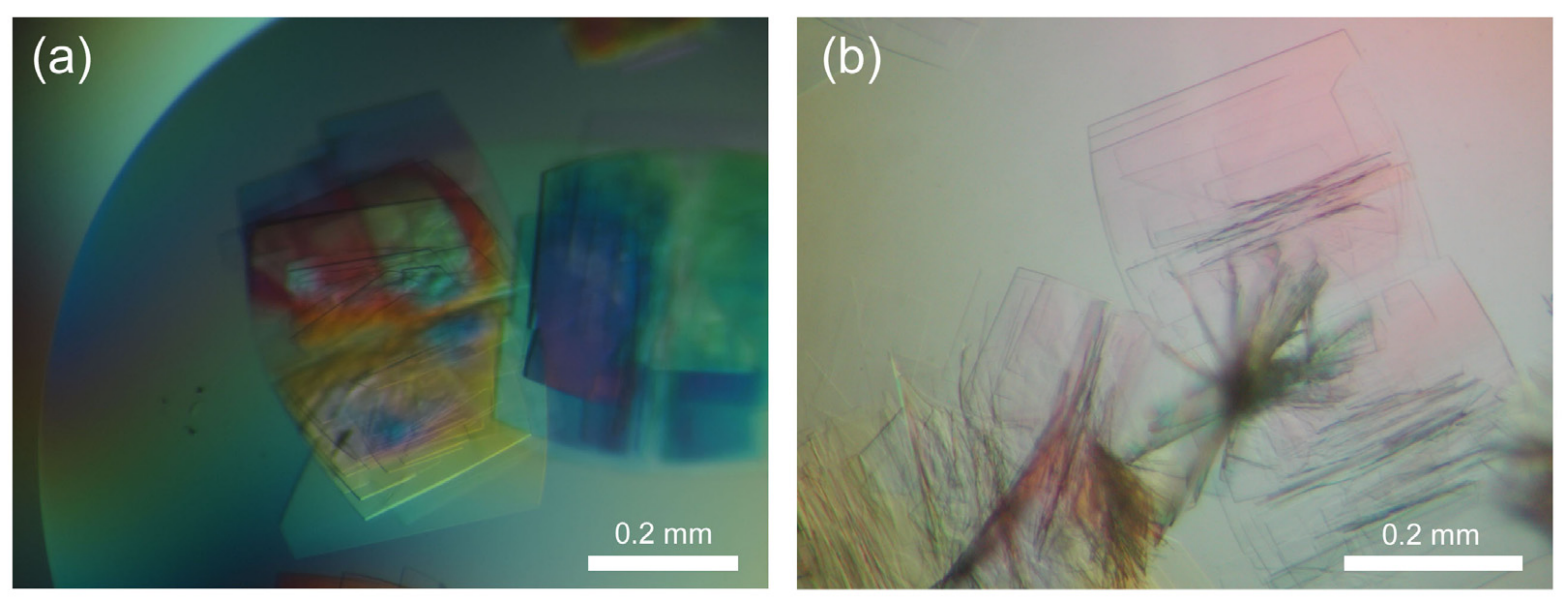
OM images of non-stained TAF-Iβ crystals in crystallization solution. OM visualized multi-layer of trapezoid or triangle crystals, with approximately 0.4 mm × 0.4 mm dimensions (**a**,**b**).

Next, we tried *in situ* observation of another protein crystal. A histone chaperone TAF-Iβ was crystallized by the sitting-drop vapor diffusion method [19]. Droplet solution (4 μL) was transferred onto a SiN film window of an ASEM dish, supplemented with 10 μL of crystallization buffer, and covered by a cover slip. Then, micro-crystals were observed in the droplet solution using ASEM without staining (Figure 8). The crystals were again darkly visualized, and features multi-layer of thin crystals with trapezoid or triangle outlines (Figure 8a). Some of the crystals were single crystals (Figure 8b). The smallest observable dimension of crystals by ASEM was about a few μm. The shape and multi-layered structure of the micro-crystals are similar to those of large TAF-Iβ crystals imaged with OM (Figure 9). Although the dimensions are larger than the wavelength of visible light, OM observation of such structure is very hard.

### 2.6. *In Situ* Observation of Non-Stained Lipase B Crystal

**Figure 10:**
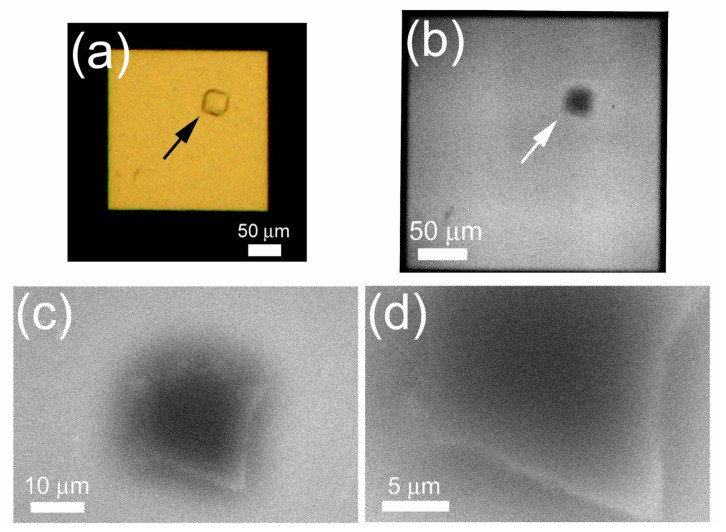
Non-stained crystals of Lipase B in crystallization solution. Lipase B was crystallized on the ASEM dish, and crystals were observed using OM (**a**) and SEM (**b**–**d**). Arrows indicate the magnified crystal.

Since the ASEM dish has an open configuration, which is different from typical crystallization plates used in the X-ray crystallography, samples should be transferred from a crystallization plate to an ASEM dish in most cases. However, such treatments may damage protein crystals. To avoid the crystal damage, we prepared a special ASEM dish with a crystallization-well. A plastic tube with 16 mm diameter and 16 mm height was glued to the standard ASEM dish to make a well (Figure 1a, left), which has the same dimensions as a well of the standard 24-well crystallization plate. Another protein, lipase B, was then crystallized by the vapor diffusion method in a crystallization-well of the special ASEM dish. After crystal formation was confirmed using OM (Figure 10a), the crystal was observed using ASEM without staining (Figure 10b–d). The lipase B crystals were observed as dark squares at a low magnification (Figure 10b) comparable to OM. At higher magnifications, the crystal appeared as an angular square with white periphery and dark center (Figure 10c,d); the appearance is similar to the non-stained lysozyme crystals.

### 2.7. Difference between Salt and Protein Crystals

**Figure 11:**
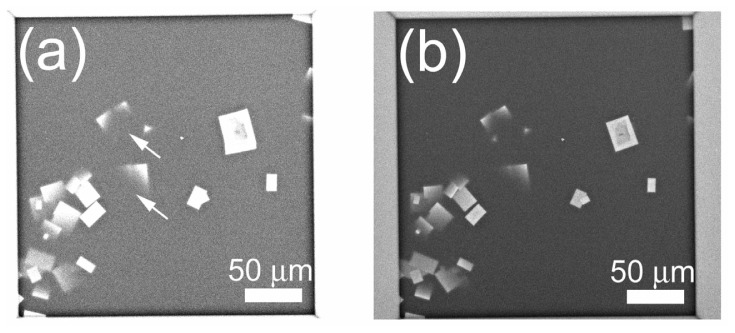
NaCl crystals in crystallization buffer. (**a**) Using EM parameters similar to Figure 7, NaCl crystals were substantially brighter than lysozyme crystals, except parts extending out of the ASEM’s observable range, namely the observable specimen thickness from the SiN film (arrows); (**b**) Non-saturated imaging of (**a**).

In protein crystallization screening, salt crystals sometimes appear in the crystallization droplet and they can be indistinguishable from protein crystals under OM. Using the ASEM, however, NaCl crystals were substantially brighter and clearer than protein crystals (Figure 11a), readily distinguishable from protein crystals (Figures 7 and 10). This is an advantage of ASEM. Crystals of ammonium sulfate in the droplet of TAF-Iβ crystallization could be easily distinguished from those of TAF-Iβ in the ASEM image due to significant difference of brightness.

Since protein and salt crystals are basically indistinguishable using visible light OM, crystals visible by OM, typically 0.05–0.1 mm, are subjected to a preliminary diffraction study with a conventional X-ray diffractometer. Ultraviolet fluorescence-based methods are also used for identifying protein crystals [20], but fluorescence intensity from crystals depends on their tryptophan content. Moreover, analysis with these methods is difficult for micro-crystals. Two optics-based methods are developed to detect protein microcrystals. Dynamic light scattering (DLS), that measures particle size from the fluctuation frequency of light scattering of the solution, has been successfully applied to crystal-screening droplet by Betzel’s group [21]. It can measure the size of protein particles in the stage of nucleation. However, it is not the visualization of crystals, thereby the morphological information is missing. Second-order non-linear optical imaging of chiral crystal (SONICC) selectively visualizes protein crystals in turbid solution, and successfully visualized 5 μm protein crystals [22]. In comparison, using the ASEM, NaCl crystals were easily distinguishable from those of protein; NaCl crystals appeared far brighter than protein crystals. Since electron backscattering depends on the atomic number and density of the sample, the stronger salt signal is attributable to its high atomic number (Na, 11; Cl, 17) compared to those of the typical protein (H, 1; C, 6; N, 7; O, 8; S, 16), and to its high density (2.18 g/cm^3^) compared to protein density (1.37 g/cm^3^) [23]. This indicates the advantage of ASEM in distinguishing protein small-crystals from salt crystals. This check can be done very quickly for relatively large crystals, because ASEM at low magnification can survey the whole window at once. Moreover, ASEM can observe non-transparent samples, which could be applied to cubic phase crystallization. The protein crystals observed here are sometimes darker, especially in the center, than the surrounding buffer. The bright edge, in contrast to the dark center, could be attributable to the BSE pathway to the detector, not only through the crystal but also through the neighboring medium, the latter of which is more efficient due to the less electron-scattering buffer composition. The dark contrast of TAF-Iβ could be attributable to the high (NH_4_)_2_SO_4_ concentration (1.43–2.85 M) in the surrounding buffer, in contrast to the moderate (NH_4_)_2_SO_4_ concentration (0.5–1 M) for lipase B or NaCl concentration (0.65–0.75 M) for lysozyme. The slightly brighter contrast of lysozyme and lipase B crystals than surrounding buffer could explain ambiguous outlines of these crystals in the ASEM images (Figures 7d and 10d). These ambiguous outlines could also be attributed to their edge positions above the electron-transparent film; resolution of ASEM images deteriorates as the height of a target object from the film increases [15].

### 2.8. ASEM Dishes for Protein Crystallization

**Figure 12:**
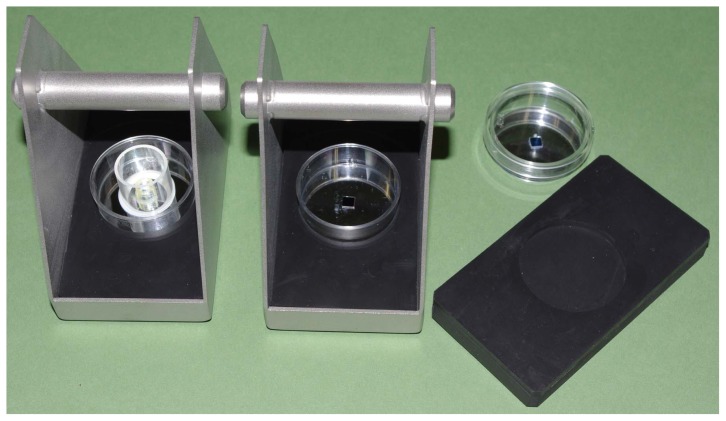
Centrifugation system for the ASEM dish with a crystallization well. Floated or suspended crystals as well as aggregations can be sedimented for inverted SEM observation. A rubber sheet with a central hole is the adaptor for the ASEM dish.

The high resolution and in-solution observation capability of ASEM [15] enabled the screening of protein micro-crystal formation. Since crystals are easily dissolved even by dislocation, *in situ* observation in crystallization buffer is important. The ASEM dish with a standard crystallization well enabled high-throughput observation of protein crystals without transfer or staining, and it can be further developed to adapt various crystallization methods for screening. Using the ASEM dish, we accomplished both the batch and sitting drop methods of crystal formation. In protein crystallization, crystals can be formed in the upper or middle of the crystallization droplet. To bring crystals or aggregation within the observable range of ASEM, which is about 2–3 μm [15] from the SiN membrane, an ASEM dish adaptor (Tomy, B407GA) for a commercial centrifuge system (Tomy, B407 12 rotor) was also developed (Figure 12).

### 2.9. Comparison of ASEM with Other High-Resolution Microscopy

*In situ* observation of crystals using traditional EM is difficult because of the vacuum in the specimen chamber. Environmental cells can protect wet samples from vacuum, but the sample space is too small, usually <15 μL, to permit a vapor diffusion method of protein crystallization. The 20 μL-capacity environmental cells for SEM [24] seem to enable observation of a droplet hanging from the capsule’s top film window, within an observable depth similar to the ASEM. However, when the protein sample crystallizes at the bottom of the droplet, observation becomes difficult.

Atomic force microscopy (AFM) accomplishes in-liquid observation of surface structure at high resolution [25] and has revealed dynamic protein crystal growth [26,27]. However, the effect of the proximity of the AFM probe on crystallization is not yet clear. The ASEM’s higher frame rate (0.15 s/frame) could complement conventional AFM.

Since the focus position (height of the ASEM dish’s SiN film from inverted SEM) is always similar, the operation of ASEM is easy. Because of its capability and structural advantages, the ASEM could be a useful new screening tool for protein crystallization. ASEM applications are expected to include not only various 3D crystallizations but also polymer chemistry and nano-science.

## 3. Experimental Section

### 3.1. Crystallization

Copper sulfate was crystallized in 10 μL copper sulfate solution (173 mg/mL, 45 °C). Crystallization was induced by evaporation and temperature decrease on an open ASEM dish. Sodium chloride was crystallized in 10 μL solution of 5 M NaCl, 200 mM sodium acetate buffer (pH 4.7) in a similar manner.

Chicken egg-white lysozyme (Sigma) was crystallized by the batch method. The protein solution (90 mg/mL, 200 mM sodium acetate buffer, pH 4.7) was mixed with the equal volume of sodium acetate buffer (200 mM, pH 4.7) containing 1.5–1.3 M NaCl on an ASEM dish, and incubated at 26 °C. Lipase B (Hampton Research) was crystallized by the sitting drop vapor diffusion method following to the manufacturer’s instructions. The ASEM dish for protein crystallization was prepared by modifying the standard ASEM dish. A cylindrical plastic tube (16 mm diameter and 16 mm height) with a glass cover on the top was glued on the standard ASEM dish (Figure 1). Lipase B protein solution (10 mg/mL) was mixed with equal volumes of 100 mM citric acid-sodium citrate buffer (pH 4.0) containing 1 M ammonium sulfate on this ASEM dish for protein crystallization, and incubated at 26 °C. TAF-Iβ protein was crystallized by the sitting-drop vapor diffusion method [19]. The droplet was prepared by mixing the equal volumes of protein solution (32 mg/mL, 20 mM Tris_HCl, 100 mM NaCl, 10mM 2-mercaptoethanol, pH 7.9) and the crystallization buffer (2.85 M ammonium sulfate, 0.1 M sodium citrate, 0.2 M potassium sodium tartrate, 30 mM MgCl_2_, pH 5.4).

### 3.2. Heavy Metal Staining of Protein Crystals

After rinsing with washing buffer (1.5 M NaCl, 200 mM sodium acetate buffer, pH 4.7), lysozyme crystals were stained with 1% PTA or 0.3% TI-Blue, Pt_4_(NH_3_)_8_(C_6_H_13_O_5_)_4_, (Nisshin-EM Ltd.) in the 2-fold diluted washing buffer for 5 min, and washed with the washing buffer. Micro-crystals were stained with the washing buffer containing 1% PTA to prevent solubilization of the crystals.

### 3.3. Imaging

The ASEM (JASM-6200, JEOL Ltd.) was used for both EM and OM (Figure 1b) except OM images of TAF-Iβ crystals taken by Discovery V8 (Zeiss). We used the standard 35 mm ASEM dish with a 100 nm thick, 0.25 × 0.25 mm silicon nitride (SiN) film window [15], and the ASEM dish with the crystallization well. Backscattered electrons were imaged using the inverted SEM operated at an electron acceleration voltage of 20 or 30 kV. It takes 80 s to take a single image (1280 × 960 pixels) at each magnification. It takes almost 28 min to observe a whole ASEM window at the magnification applied in Figures 6C,7C,8A (×2000), which is enough for the observation of a few μm-crystals. The electron dose for these observations is 3 electrons/Å^2^ or less, which is less than 1/7 of the dose of 20 electrons/Å^2^ for cryo-TEM single particle reconstruction of sodium channel protein [7]. Copper sulfate crystallization was video-recorded at a frame rate of 0.15 s, with averaging set at four, and was monitored as time-course imaging.

## 4. Conclusions

While ASEM, an easy-to-use microscope with inverted SEM and normal OM, was originally developed for cell and tissue observation, it can observe micro-crystals in crystallization buffer using a newly developed crystallization dish that has an electron-transparent thin membrane at the bottom. ASEM should be useful for the initial screening of protein crystallization, because it can give clear images of micro-crystals of less than a few μm without staining, enabling us to start optimization of the crystallization conditions from a very early stage of the screening. Furthermore, it was possible to distinguish protein crystals from salt crystals due to significant differences in their contrasts.
